# Giant fusiform internal jugular venous aneurysmal dilatation diagnosed coincidentally during right carotid endarterectomy combined with coronary bypass surgery

**DOI:** 10.1186/1749-8090-8-S1-P55

**Published:** 2013-09-11

**Authors:** H Cakir, U Yetkin, N Karakas, A Gurbuz

**Affiliations:** 1 Department of Cardiovascular Surgery, Izmir Katip Celebi University Ataturk Training and Research Hospital, Izmir, Turkey

## Background

Venous aneuysmal dilatation or flebectasia (venous ectasia) or venous congenital cyst is a benign pathology which represents an abnormal dilatation of a vein and it is frequently seen with the improvements in non-invasive diagnostic methods of laryngeal and cervical diseases.

## Methods

Our case was a 68 year-old male. On the coronary angiography, which was performed with the complaint of chest pain, three-vessel disease was detected and the patient was hospitalized for surgery. Routine bilateral carotid colored duplex ultrasonography revealed 70% stenosis in the proximal segment of right internal carotid artery (ICA). Provided that, he was undertaken to carotid angiography and 75% stenosis was seen in the right ICA.

## Results

In the light of these findings, right carotid endarterectomy was planned electively and simultaneously with coronary by-pass grafting (CABG) operation under general anesthesia. As right sternocleidomastoid muscle was carefully dissected for exploration of common carotid artery (CCA), huge fusiform aneurysmal internal jugular vein was confronted. CCA and its’ branches were carefully explored, and then carotid endarterectomy and CABG were performed simultaneously. He was taken to intensive care unit then to service follow-up without any complications.

## Conclusions

Venous aneurismal dilatation or flebectasia was thought to be congenital as seen rarely in adulthood, and as it doesn’t cause any symptom diagnosed coincidentally thus. Conservative clinical follow-up is required in this situation. Rupture of dilated vein is a hypothetic complication. Surgery may be needed in case of symptomatic patients, cosmetic reasons, thrombotic occasions and rupture of the vein.

